# A cross-sectional study to investigate associations between flooring substrates and prevalence of limb and paw abnormalities of dogs housed in commercial breeding facilities

**DOI:** 10.3389/fvets.2025.1466390

**Published:** 2025-02-19

**Authors:** Judith Stella, Paulo Gomes, Traci Shreyer, Candace Croney

**Affiliations:** ^1^Department of Comparative Pathobiology, Purdue University, West Lafayette, IN, United States; ^2^Department of Veterinary Clinical Sciences, Purdue University, West Lafayette, IN, United States; ^3^Center for Animal Welfare Science, Purdue University, West Lafayette, IN, United States; ^4^Department of Animal Science, Purdue University, West Lafayette, IN, United States

**Keywords:** welfare, breeding dogs, flooring substrates, paw health, limb health

## Abstract

Understanding the impact of environmental and management factors on the health and behavior of dogs housed in commercial breeding (CB) facilities is critical to their welfare. The specific aims of the study were to assess (a) associations between combinations of flooring substrates commonly used in CB kennels with foot, elbow, or hock abnormalities such as pododermatitis, calluses, or interdigital furuncle and (b) the impact of flooring substrate on dog cleanliness. Dogs (*N* = 373) from CB facilities (*N* = 20), housed on combinations of concrete, gravel, and diamond-coated expanded metal were assessed. A veterinary dermatologist examined each dog's paw, toenails, elbows, hocks, body condition, and overall cleanliness. Identified conditions included wet paws (12.6%), calluses (11.26%), erythema (6.97%), and matted paw fur (6.17%). Mixed-effects logistic regression models identified an effect of sex and wet paws (OR 6.08, CI 1.23, 29.92, *p* = 0.03) and age with matted paw fur (OR 1.52, CI 1.12, 2.07, *p* = 0.007). A few conditions were identified, including pododermatitis, hygromas, and interdigital furuncles, where management alterations might result in improved outcomes and welfare states for dogs in CB facilities.

## 1 Introduction

Concerns for the health and welfare of dogs housed in large commercial breeding (CB) facilities in the US exist, including aspects of the housing environment such as the quantity and complexity of space provided, socialization and management practices, and veterinary care provided. The stress associated with poor housing and rearing conditions has been associated with negative health and behavior outcomes (1, 2). Welfare concerns specific to dogs housed in kennels include limited opportunities for early socialization (3–5), limits on environmental control (6, 7), restricted space (8, 9), opportunities to exercise (10), social opportunities (11), and environmental complexity (11–13). Dogs in CB facilities may be exposed to additional stress due to minimal or poor human-animal interactions (14), and concerns related to transport to and from the kennel environment for sale or re-homing of retired dogs (14, 15). Any or all such factors can contribute to physiological and behavioral stress in dogs. Thus, evaluation of these factors is critical to ensuring that dogs in CB facilities are provided with a housing and rearing environment that promotes optimal health, development, and socialization.

Little research has aimed at investigating the impact of specific aspects of the housing environment on dog welfare, such as flooring. The flooring substrates used in the primary housing area of dogs maintained in CB kennels have elicited concerns relating to their safety for dogs and related potential effects on dogs' health and well-being (16, 17). There is currently no federal regulation restricting the use of any flooring substrate, while restrictions vary by state with prohibitions on the use of some surfaces [e.g., gravel, wire-strand, and diamond-coated expanded metal (DECM)] due to concerns around the potential injury to dogs that are housed on them and the ability of caretakers to effectively clean and sanitize the flooring. Paw and leg conditions that may be affected by flooring substrates include pododermatitis, callus formation on hocks and elbows, and the development of interdigital furunculosis (16).

Pododermatitis is of particular significance as it is an inflammation of the feet that manifests clinically as interdigital dermatitis, pedal folliculitis and furunculosis, and interdigital furunculosis. Causes are numerous and may include trauma, foreign bodies, contact with irritants, hypersensitivity disorders, parasites, infections (e.g., yeast, bacteria, or fungi), immune-mediated skin diseases, and abnormal foot conformation (18, 19). Increased friction between the feet and rough surfaces, such as gravel or vegetation, can lead to trauma, and excessive licking associated with allergic dermatitis which can result in self-trauma. Trauma to the haired skin of the palmar and plantar webs is involved in the pathogenesis of interdigital follicular cysts (20).

The formation of a callus, a localized hyperplastic skin lesion caused by repeated friction or pressure, may be a factor that is associated with long-term housing on some types of flooring substrates. Calluses are well-demarcated, hyperplastic, often hyperpigmented plaques that develop over bony prominences called pressure points. The most common site for the development of a callus is the elbow, followed by the hocks, sternum, and hips (21). While dogs of any size and breed can develop calluses, large dogs such as the Great Dane, St. Bernard, Newfoundland, and Irish Wolfhound appear to be overrepresented in clinical diagnoses. In these breeds, calluses most often occur on the elbows and hocks. In contrast, short-legged dogs, such as Dachshunds or dogs with deep-seated chests, such as the Pointer, Boxer, and Doberman Pincher, tend to develop calluses on the sternum. Obesity also predisposes dogs to the development of calluses over pressure points. Secondary infections are not uncommon, with severe cases progressing to callus pyoderma (18).

Interdigital furuncle is a localized infection or abscess between the toes of dogs, most commonly affecting the front paws. These are painful, pus-filled sacs located in the webbing between the toes as a result of a deep bacterial infection (22). Predisposing factors include short hair, excess weight, and altered weight bearing. Breeds that have short, rough hair between the toes and/or increased webbing, such as the Chinese Shar-Pei, Labrador Retriever, and Bulldog, are predisposed to the condition. It is a multifactorial condition with primary, predisposing, and perpetuating causes that lead to secondary infections. Common factors leading to inflammation include atopic dermatitis, food allergies, ingrown hairs, and abnormal conformation.

Despite the possible connection between the development of these adverse health conditions and the type of flooring on which dogs are maintained, few studies have explored the existence of such associations, especially on-site at US CB kennels.

Stella et al. (17) reported the results of a study examining dogs' physical health and cleanliness when housed on three common flooring types [diamond-shaped coated expanded metal (DCEM), polypropylene (POLY), and concrete (CON)] in four CB facilities in Indiana, US. No significant paw, elbow, or hock conditions were identified. Additionally, dog and kennel cleanliness was maintained on all substrates assessed. The focus of the present study was to elaborate upon this earlier study with more diverse flooring types, the inclusion of certain outdoor flooring surfaces that were not investigated in the initial study, and a larger study population. Dogs examined were housed on three common combinations of flooring substrates used in CB facilities located in Indiana and Illinois: (1) concrete inside and outside (CON/CON); (2) diamond-coated expanded metal inside and concrete outside (DECM/CON); and (3) concrete inside, gravel outside (CON/GRAV). The specific aims of the study were to assess (a) associations between combinations of flooring substrates commonly used in CB kennels with foot, elbow, or hock abnormalities such as pododermatitis, calluses, or interdigital furuncle and (b) the effects of flooring substrate on dog cleanliness.

## 2 Materials and methods

### 2.1 Facilities and subjects

Twenty Amish-owned CB kennels volunteered to participate in the study. Visits for data collection were scheduled during regular business hours (09:00–17:00). A total of 373 adult dogs (mean age 2.94 ± 1.58, range 1–8 years) were examined, including 287 females and 80 males (sex was not recorded for 6 dogs), representing 42 breeds (Table 1). Female dogs in the last 2 weeks of gestation and those nursing puppies were excluded. All dogs at the facility that met the inclusion criteria were examined. Dogs were single-, pair-, or group-housed in a kennel that consisted of an indoor run with access to an outdoor run. The flooring substrates assessed in this study were those commonly used in CB facilities located in Indiana and Illinois: (1) sealed concrete or tile inside and sealed concrete outside (CON/CON), (2) diamond-shaped coated expanded metal inside and sealed concrete outside (DCEM/CON), and (3) sealed concrete inside and gravel outside (CON/GRAV). The pens included feeders and automatic water systems or buckets sufficient for the number of dogs in each pen. Some breeders provided enrichment items such as a toy or chew items (e.g., cow hoof) but none contained beds or bedding.

**Table 1:** Summary of age, breed, and flooring substrate at each facility.

**Facility**	**Age**	**Breeds (*N*)**	**Flooring substrate (*N*)**
	**Mean (SD)**		
1	3.58 (1.62)	French bulldogs (17)	CON/CON (16), CON/GRAVEL (1)
2	2.75 (1.34)	Miniature Australian Shepherds (6), Lhasa Apso (7), Scottish Terriers (3)	DCEM/CON (16)
3	3.58 (1.75)	Italian Greyhound (16), Maltese (2)	CON/GRAVEL (18)
4	4.35 (1.53)	Pomsky (1), Standard Poodle (1), Australian Shepherd (10), Golden Retriever (7), Siberian Husky (3), German Shepherd (1)	CON/CON (23)
5	2.96 (1.59)	Standard Poodle (1), Golden Retriever (2), Basset Hound (3), Bernese Mountain Dog (4), Miniature Goldendoodle (2), Pembroke Welsh Corgi (11)	DCEM/CON (23)
6	3.04 (1.41)	Standard Poodle (1), Golden Retriever (2), Saint Bernard (4), Cockapoo (1), Cocker Spaniel (5)	CON/GRAVEL (12), DCEM/CON (1)
7	3.0 (2.35)	Golden Retriever (5)	CON/CON (5)
8	2.8 (1.39)	Siberian Husky (6), Boxer (2), Great Dane (10), Samoyed (2)	CON/GRAVEL (20)
9	1.60 (1.07)	Shih Tzu (24)	CON/GRAVEL (24)
10	3.58 (1.82)	Lhasa Apso (1), Shih Tzu (2), Pomeranian (1), Havanese (8)	CON/CON (12)
11	1.84 (0.92)	French Bulldog (7), Miniature Poodle (4), Boston Terrier (8), Victorian Bulldog (1), Cavalier King Charles Spaniel (2)	CON/GRAVEL (22)
12	2.62 (1.12)	French Bulldog (8), Victorian Bulldog (1), English Bulldog (3), Olde Bulldog (3), Shiba Inu (3), Soft-coated Wheaten Terrier (2), Pug (1)	CON/CON (21)
13	2.03 (1.02)	Maltese (6), Miniature Poodle (2), Morkie (1), Yorkshire Terrier (1), Dachshund (8)	CON/GRAVEL (18)
14	2.89 (0.74)	Maltese (3), Miniature Poodle (2), Shih Tzu (2), Pomeranian (6)	CON/GRAVEL (20)
15	3.61 (1.99)	Maltese (9), Miniature Poodle (9), Papillon (4)	DCEM/CON (22)
16	2.73 (1.30)	Miniature Australian Shepherds (2), Pembroke Welsh Corgi (5), Weimaraner (8), Soft-coated Wheaten Terrier (5)	DCEM/CON (20)
17	3.3 (1.63)	Maltese (7), Morkie (1), Yorkshire Terrier (8), Miniature Pinscher (4)	DCEM/CON (20)
18	2.5 (1.13)	French Bulldog (5), Miniature Poodle (4), Golden Retriever (2), Miniature Goldendoodle (2), Pembroke Welsh Corgi (2), Cocker Spaniel (1), Pomeranian (1), Shiba Inu (2), Labrador Retriever (2)	CON/CON (21)
19	3.69 (1.57)	Miniature Australian Shepherds (1), Standard Poodle (3), Golden Retriever (8), Bernese Mountain Dog (1), Miniature Goldendoodle (4), Shih Tzu (1), Cavalier King Charles Spaniel (1), Bernedoodle (2)	CON/CON (21)
20	3.06 (1.95)	French Bulldog (3), Pomsky (2), Siberian Husky (8), Pomeranian (2), English Bulldog (2)	CON/CON (17)
Total	2.94 (1.58)	42 breeds (373)	

### 2.2 Experimental procedure

Data were collected between May 30 and June 21, 2019, at least 2 h after routine husbandry procedures. The dogs were brought to a designated area of the kennel facility one at a time and examined by a board-certified veterinary dermatologist. The physical exam included an assessment of each paw, the toenails, elbows, hocks, body condition, and overall dog cleanliness. The interdigital areas, pads, and toenails of each paw and the limbs, including the elbows and hocks, were assessed for evidence of any abnormalities, including matting and wet paws, as defined in Table 2.

**Table 2:** Definition of paw, elbow, and hock conditions assessed.

Abrasion	Scraped area on the skin or on a mucous membrane, resulting from injury or irritation.
Acral lick dermatitis	Lesion, typically on distal portions of one or more limbs. resulting from excessive licking. May be red and/or swollen, irritated, bleeding, eventually becoming a thick, firm, plaque.
Alopecia	Loss of hair, partial or complete, in areas where it normally grows.
Callus	Thickened and hardened area of skin resulting from excess friction.
Cyst	Closed capsule or sac-like structure, typically filled with fluid, semi-solid or gaseous material, similar to a blister.
Erythema	Superficial reddening of the skin, usually in patches, resulting from injury or irritation causing dilatation of the blood capillaries.
Hygroma	Fluid-filled swelling caused by repeated trauma.
Hyperkeratosis	Hypertrophy of the horny layer of the skin, or any disease characterized by the same.
Hyperplasia	Abnormal increase in the volume of tissue caused by formation and growth of new normal cells.
Interdigital furuncles	Painful nodular lesions located in the interdigital webs, histologically representing areas of nodular pyogranulomatous inflammation.
Matted	Hair or fur tangled into a thick mass.
Nodule	A small swelling or aggregation of cells, especially an abnormal one.
Pododermatitis	A condition characterized by inflammation of the dermal tissue of the paws.
Sore	Any raw or painful place
Wound	An injury to living tissue caused by a cut, blow, or other impact, in which the skin is broken.

A body condition score (BCS) was recorded at the time of the physical examination using a 5-point scale as follows: 1 = emaciated; 2 = thin; 3 = ideal; 4 = mildly overweight; 5 = obese.

A body cleanliness score (BCLS) was recorded based on a five-point scale developed by the experimenters as follows: 1 = 0%: No debris present on the dog; 2 = 1–25%: Debris present on the paws only; 3 = 26–50%: Debris on the legs, chest, and/or abdomen; 4 = 51–75%: Debris covering most of the dog except his/her head and neck; 5 >76%: Debris present on all parts of the dog including the neck and head.

Toenail length was scored using a five-point scale developed by the experimenters as follows: 1 = Normal length; 2 = Slightly overgrown; 3 = Overgrown, but not curling; 4 = Beginning to curl, ± growing into the pads, ± broken or missing nails; and 5 = Curling, growing into the pad, cracked and/or missing nails. Any abnormalities of the toenails (e.g., onycholysis, onychoschizia, onychorrhexis, onychodystrophy) were also recorded.

### 2.3 Statistical analysis

Descriptive statistics (e.g., means, standard deviation) were used to summarize the findings. Outcome variables of interest that were observed in >2% of the study population were retained and mixed-effects logistic regression models were constructed. The outcome variables retained were calluses, erythema, matted hair (paws), pododermatitis, and wet paws. Logistic regression models contained the predictor variables sex, age, BCS, breed, and flooring type with facility as the group variable. A backward-stepwise model selection method was applied, where fixed effects were sequentially removed starting with the least significant term, until only significant predictors were left in the model. The model that best fit the data was then selected by comparing Akaike information criteria (AIC) values between models. A separate model was run for each of the binomial health outcomes retained. Analysis was performed using commercially available statistical software (StataCorp LP, College Station, TX, USA).

## 3 Results

### 3.1 Body condition

The mean body condition score was 3.05, ranging from 2 to 4, with 328 of 373 dogs being scored 3 for ideal BCS.

### 3.2 Body cleanliness

Most dogs (99%, 369/374) were given a score of 1 or 2. Four dogs, all housed on CON/CON, were observed with more areas of their bodies than just their paws being noted as dirty (2 dogs scored 3, 1 dog scored 4, 1 dog scored 5).

### 3.3 Nail condition

No significant nail conditions were identified; 96% (131/136) of dogs housed on CON/CON were given a score of 1 and 4% (5/136) were given a score of 2; 96% (130/135) of dogs housed on CON/GRAV were given a score of 1, 3% (4/135) were given a score of 2, and 1% (1/135) were given a score of 3; 97% (99/102) of dogs housed on DCEM/CON were given a score of 1 and 3% (3/102) were given a score of 2.

### 3.4 Elbow, hock, and paw conditions

**Figure 1:**
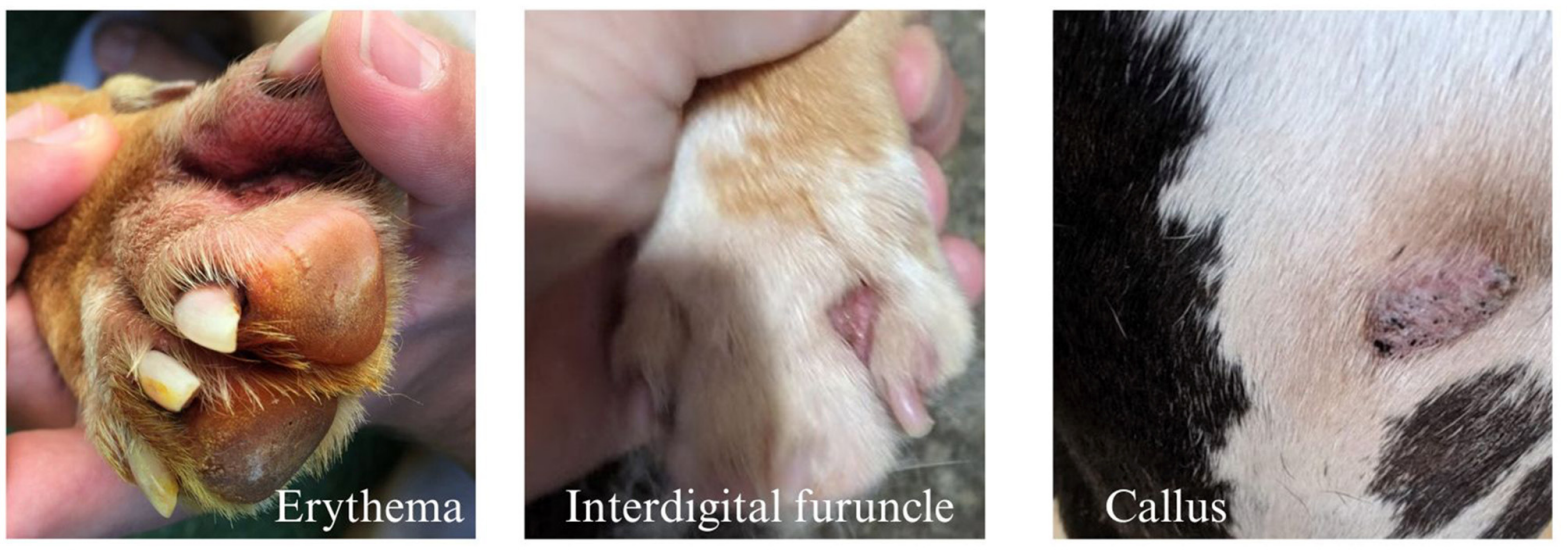
Photo examples of some conditions identified. From left to right, an example of erythema, an interdigital furuncle, and an elbow callus.

The most common conditions identified were wet paws (12.6%), calluses (11.26%), erythema (6.97%), matted fur between the paw pads (6.17%), pododermatitis (4.29%), and interdigital furuncles (2.68%) (Table 3, Figure 1). Hygromas were identified in large and giant breed dogs (*n* = 5) and ID furuncles were primarily observed in bulldog breeds (9/10 observations) (Table 4). Results of the mixed-effects logistic regression models identified a statistically significant effect of sex and wet paws (OR 6.08, CI 1.23, 29.92, *p* = 0.03, males 15% vs. females 11.9%) and age with matted hair between paw pads (OR 1.52, CI 1.12, 2.07, *p* = 0.007) (Table 5). The mean age of dogs with matted paws was 3.72 ± 1.55, range 1–6 vs. 2.89 ± 1.55, range 1–8 for dogs without matted paws. No significant effect of flooring substrate, BCS, or breed was identified for any of the conditions of interest.

**Table 3:** Elbow, hock, paw conditions identified for each flooring substrate.

**Condition**	**% Affected**	**CON/CON (*N*/%)**	**CON/GRAV (*N*/%)**	**DCEM/CON (*N*/%)**
Abrasion	0.27	0/0	1/0.7	0/0
Acral lick dermatitis	0.27	0/0	1/0.7	0/0
Alopecia	1.61	2/1.47	4/2.96	0/0
Calluses	11.26	23/17	15/11	4/3.9
Cyst	0	0/0	0/0	0/0
Erythema	6.97	20/14.7	5/3.7	1/0.98
Hygroma	1.34	4/2.94	0/0	1/0.98
Hyperkeratosis	1.34	3/2.2	2/1.48	0/0
Hyperplasia	0.8	1/0.74	2/1.48	0/0
ID Furuncles	2.68	6/4.4	4/3	0/0
Matted	6.17	5/6.2	7/3.7	11/10.8
Nodule	0	0/0	0/0	0/0
Pododermatitis	4.29	12/8.8	2/1.5	2/2
Sore	0.27	0/0	1/0.7	0
Wet paws	12.60	40/29	2/1.5	5/4.9
Wound	0.27	0/0	1/0.7	0/0

Number of dogs housed on each combination: CON/CON = 136 dogs, CON/GRAV = 135 dogs, DCEM/CON = 102 dogs.

**Table 4:** By breed and flooring type, conditions that were observed in >1% of the dogs.

**Breed**	**Flooring**	**Alopecia**	**Callus**	**Erythema**	**Hygroma**	**Hyperkeratosis**	**ID Furuncles**	**Matted**	**Pododermatitis**	**Wet paws**
Australian Shepherd (*n =* 10)	CON/CON	0	1	1	0	1	0	0	0	1
	CON/GRAV	0	0	0	0	0	0	0	0	0
	DCEM/CON	0	0	0	0	0	0	0	0	0
Bassett Hound (*n =* 3)	CON/CON	0	0	0	0	0	0	0	0	0
	CON/GRAV	0	0	0	0	0	0	0	0	0
	DCEM/CON	0	0	0	0	0	0	0	0	0
Bernese Mountain Dog (*n =* 5)	CON/CON	0	0	0	0	0	0	0	1	1
	CON/GRAV	0	0	0	0	0	0	0	0	0
	DCEM/CON	0	0	0	0	0	0	0	0	0
Bernadoodle (*n =* 2)	CON/CON	0	0	0	0	0	0	0	2	2
	CON/GRAV	0	0	0	0	0	0	0	0	0
	DCEM/CON	0	0	0	0	0	0	0	0	0
Boston Terrier (*n =* 8)	CON/CON	0	0	0	0	0	0	0	0	0
	CON/GRAV	0	0	0	0	0	0	0	0	0
	DCEM/CON	0	0	0	0	0	0	0	0	0
Bulldog (*n =* 5)	CON/CON	0	0	0	0	0	2	0	0	0
	CON/GRAV	0	0	0	0	0	0	0	0	0
	DCEM/CON	0	0	0	0	0	0	0	0	0
Boxer (*n =* 2)	CON/CON	0	0	0	0	0	0	0	0	0
	CON/GRAV	0	0	0	0	0	0	0	0	0
	DCEM/CON	0	0	0	0	0	0	0	0	0
Cavalier King Charles Spaniel (*n =* 3)	CON/CON	0	0	0	0	0	0	0	0	1
	CON/GRAV	0	0	0	0	0	0	0	0	0
	DCEM/CON	0	0	0	0	0	0	0	0	0
Cockapoo (*n =* 1)	CON/CON	0	0	0	0	0	0	0	0	0
	CON/GRAV	0	0	0	0	0	0	0	0	0
	DCEM/CON	0	0	0	0	0	0	0	0	0
Cocker Spaniel (*n =* 6)	CON/CON	0	0	0	0	0	0	0	0	0
	CON/GRAV	0	0	0	0	0	0	0	1	0
	DCEM/CON	0	0	0	0	0	0	0	0	0
Dachshund (*n =* 8)	CON/CON	0	0	0	0	0	0	0	0	0
	CON/GRAV	0	0	0	0	0	0	0	0	0
	DCEM/CON	0	0	0	0	0	0	0	0	0
French Bulldog (*n =* 40)	CON/CON	1	15	16	0	1	2	0	1	16
	CON/GRAV	0	0	0	0	0	3	0	0	0
	DCEM/CON	0	0	0	0	0	0	0	0	0
German Shepherd Dog (*n =* 1)	CON/CON	0	1	1	0	0	0	0	0	0
	CON/GRAV	0	0	0	0	0	0	0	0	0
	DCEM/CON	0	0	0	0	0	0	0	0	0
Golden Retriever (*n =* 26)	CON/CON	0	1	1	0	0	0	2	1	10
	CON/GRAV	0	0	1	0	0	0	0	0	0
	DCEM/CON	0	0	0	0	0	0	0	0	0
Great Dane (*n =* 10)	CON/CON	0	0	0	0	0	0	0	0	0
	CON/GRAV	2	6	1	2	0	0	0	0	0
	DCEM/CON	0	0	0	0	0	0	0	0	0
Havanese (*n =* 8)	CON/CON	0	0	0	0	0	0	1	0	0
	CON/GRAV	0	0	0	0	0	0	0	0	0
	DCEM/CON	0	0	0	0	0	0	0	0	0
Italian Greyhound (*n =* 16)	CON/CON	0	0	0	0	0	0	0	0	0
	CON/GRAV	1	8	1	0	0	0	0	0	0
	DCEM/CON	0	0	0	0	0	0	0	0	0
Labrador Retriever (*n =* 2)	CON/CON	0	1	0	0	0	0	0	0	0
	CON/GRAV	0	0	0	0	0	0	0	0	0
	DCEM/CON	0	0	0	0	0	0	0	0	0
Lhasa Apso (*n =* 8)	CON/CON	0	0	0	0	0	0	1	0	0
	CON/GRAV	0	0	0	0	0	0	1	0	0
	DCEM/CON	0	0	0	0	0	0	0	0	0
Maltese (*n =* 18)	CON/CON	0	0	0	0	0	0	0	0	0
	CON/GRAV	0	0	0	0	0	0	0	0	0
	DCEM/CON	0	0	0	0	0	0	3	0	0
Miniature Australian Shepherd (*n =* 9)	CON/CON	0	0	0	0	0	0	0	1	1
	CON/GRAV	0	0	0	0	0	0	0	0	0
	DCEM/CON	0	0	1	0	0	0	0	0	0
Miniature Goldendoodle (*n =* 8)	CON/CON	0	0	0	0	0	0	0	2	4
	CON/GRAV	0	0	0	0	0	0	0	0	0
	DCEM/CON	0	0	0	0	0	0	0	0	0
Miniature Pinscher (*n =* 4)	CON/CON	0	0	0	0	0	0	0	0	0
	CON/GRAV	0	0	0	0	0	0	0	0	0
	DCEM/CON	0	1	0	0	0	0	0	0	0
Morkie (*n =* 3)	CON/CON	0	0	0	0	0	0	0	0	0
	CON/GRAV	0	0	0	0	0	0	0	0	0
	DCEM/CON	0	0	0	0	0	0	0	0	0
Olde English Bulldog (*n =* 3)	CON/CON	1	0	1	0	0	1	0	0	0
	CON/GRAV	0	0	0	0	0	0	0	0	0
	DCEM/CON	0	0	0	0	0	0	0	0	0
Papillion (*n =* 1)	CON/CON	0	0	0	0	0	0	0	0	0
	CON/GRAV	0	0	0	0	0	0	0	0	0
	DCEM/CON	0	0	0	0	0	0	0	0	0
Pembroke Welsh Corgi (*n =* 18)	CON/CON	0	0	0	0	0	0	0	0	0
	CON/GRAV	0	0	0	0	0	0	0	0	0
	DCEM/CON	0	0	0	0	0	0	0	0	2
Pomeranian (*n =* 19)	CON/CON	0	0	0	0	0	0	0	0	0
	CON/GRAV	0	0	0	0	0	0	0	0	0
	DCEM/CON	0	0	0	0	0	0	0	0	0
Pomsky (*n =* 3)	CON/CON	0	0	0	0	0	0	0	0	0
	CON/GRAV	0	0	0	0	0	0	0	0	0
	DCEM/CON	0	0	0	0	0	0	0	0	0
Poodle (*n =* 18)	CON/CON	0	0	0	0	0	0	1	2	3
	CON/GRAV	0	0	0	0	0	0	0	0	0
	DCEM/CON	0	0	0	0	0	0	0	0	1
Pug (*n =* 1)	CON/CON	0	0	0	0	0	0	0	0	0
	CON/GRAV	0	0	0	0	0	0	0	0	0
	DCEM/CON	0	0	0	0	0	0	0	0	0
Saint Bernard (*n =* 4)	CON/CON	0	0	0	0	0	0	0	0	0
	CON/GRAV	0	0	0	2	0	0	0	0	0
	DCEM/CON	0	0	0	0	0	0	2	0	0
Samoyed (*n =* 2)	CON/CON	0	0	0	0	0	0	0	0	0
	CON/GRAV	0	0	0	0	0	0	0	0	0
	DCEM/CON	0	0	0	0	0	0	0	0	0
Schipperke (*n =* 4)	CON/CON	0	0	0	0	0	0	0	0	0
	CON/GRAV	0	0	0	0	0	0	0	0	0
	DCEM/CON	0	0	0	0	0	0	0	0	1
Scottish Terrier (*n =* 3)	CON/CON	0	0	0	0	0	0	0	0	0
	CON/GRAV	0	0	0	0	0	0	0	0	0
	DCEM/CON	0	0	0	0	0	0	0	0	3
Shiba Inu (*n =* 5)	CON/CON	0	0	0	0	0	0	0	0	0
	CON/GRAV	0	0	0	0	0	0	0	0	0
	DCEM/CON	0	0	0	0	0	0	0	0	0
Shih Tzu (*n =* 38)	CON/CON	0	0	0	0	0	0	0	1	1
	CON/GRAV	1	1	1	0	1	1	5	1	2
	DCEM/CON	0	0	0	0	0	0	2	0	1
Siberian Husky (*n =* 17)	CON/CON	0	4	0	0	1	0	0	1	0
	CON/GRAV	0	0	1	0	1	0	0	0	0
	DCEM/CON	0	0	0	0	0	0	0	0	0
Soft-coated Wheaten Terrier (*n =* 7)	CON/CON	0	0	0	0	0	0	0	0	0
	CON/GRAV	0	0	0	0	0	0	0	0	0
	DCEM/CON	0	0	0	0	0	0	1	2	0
Victorian Bulldog (*n =* 2)	CON/CON	0	0	1	0	0	1	0	0	0
	CON/GRAV	0	0	0	0	0	0	0	0	0
	DCEM/CON	0	0	0	0	0	0	0	0	0
Weimaraner (*n =* 8)	CON/CON	0	0	0	0	0	0	0	0	0
	CON/GRAV	0	0	0	0	0	0	0	0	0
	DCEM/CON	0	3	0	1	0	0	0	0	0
Yorkshire Terrier (*n =* 14)	CON/CON	0	0	0	0	0	0	0	0	0
	CON/GRAV	0	0	0	0	0	0	0	0	0
	DCEM/CON	0	0	0	0	0	0	4	0	0

**Table 5:** Results of mixed-effect logistic regression models for paw, elbow, and hock conditions.

**Outcome**	**Predictor**	**OR**	**95% CI**	***P*-value**
Wet paws	Sex	6.08	1.23, 29.92	***0.03***
	DCEM/CON	Referent	N/A	N/A
	CON/GRAV	0.0007	0, 6,314	0.38
	CON/CON	26.99	0.001, 668,578	0.52
Pododermatitis	Breed	1.03	0.98, 1.04	0.16
	DCEM/CON	Referent	N/A	N/A
	CON/GRAV	1.24	0.047, 32.44	0.89
	CON/CON	5.71	0.27, 120.8	0.26
Erythema	Sex	3.14	0.66, 14.88	0.15
	Age	0.83	0.52, 1.33	0.19
	BCS	14.4	2.40, 86.45	***0.004***
	DCEM/CON	Referent	N/A	N/A
	CON/GRAV	11.03	0.10, 1169	0.31
	CON/CON	36.6	0.37, 3607	0.12
Matted	Age	1.52	1.12, 2.07	***0.007***
	Breed	0.99	0.95, 1.05	0.85
	BCS	0.40	0.13, 1.24	0.11
	DCEM/CON	Referent	N/A	N/A
	CON/GRAV	0.72	0.1, 5.14	0.74
	CON/CON	0.33	0.04, 2.55	0.29
Callus	Age	1.14	0.88, 1.48	0.32
	BCS	1.97	0.64, 6.09	0.24
	DCEM/CON	Referent	N/A	N/A
	CON/GRAV	2.59	0.13, 50.92	0.53
	CON/CON	6.91	0.397, 120.16	0.19

Bold and italic values indicate statistically significant results.

## 4 Discussion

In agreement with an earlier study (17), no significant associations between the flooring substrates studied and the prevalence of paw, elbow, or hock conditions, dog cleanliness, or body condition were identified. The body condition of each dog was collected to determine any possible relationship with the development of adverse paw or limb conditions in dogs housed on different flooring substrates. No significant association between BCS and the prevalence of paw, elbow, or hock conditions was identified. This is likely due to the infrequency of any of the conditions of interest assessed in this study and the optimal BCS of dogs. Further research is therefore needed to determine if an association exists between flooring substrate, BCS, and paw or limb health.

A few areas were identified where changes in management might lead to improved overall paw and limb health. First, more dogs housed on CON/CON were observed with wet paws, erythema, and pododermatitis than on any other flooring types or combinations. Pododermatitis is a multifactorial condition that involves predisposing (e.g., short hairs, excess weight, and abnormal conformation), primary (e.g., atopic dermatitis, food-induced atopic dermatitis, and abnormal conformation), secondary (e.g., superficial or deep yeast and bacterial infections), and perpetuating factors (18, 23). Opportunistic yeast and bacterial infections may progress to pedal folliculitis and furunculosis. Perpetuating factors will delay the resolution of pododermatitis and usually include hyperplastic lesions to the interdigital skin and footpads because of trauma and chronic inflammation (18). In addition, weight bearing on haired skin, ingrown hairs, and sinus tracts also act as perpetuating factors (23), so overweight short-haired dogs with conformational issues of the feet are at increased risk of developing pododermatitis-related interdigital furunculosis. In the current study, most dogs that developed pododermatitis had wet paws when examined and were housed on concrete flooring. It is likely that concrete takes longer to dry after sanitation, retaining moisture for extended periods of time. Excessive moisture can predispose to maceration of the stratum corneum and create a microenvironment favorable for secondary bacterial and yeast infections leading to pododermatitis. Drying the flooring after routine cleaning may decrease the risk of pododermatitis for dogs housed on concrete or other solid flooring substrates.

Secondly, hygromas were identified in several giant breed dogs housed on concrete. Hygroma is a condition that is almost always associated with large dogs because of repetitive trauma to the skin overlying the olecranon. The lesion is characterized by a fibrous connective tissue capsule filled with serum fluid (24). In accordance with the current literature, hygromas were only identified in giant breed dogs housed on concrete flooring. Treatment of this condition can be difficult, with conservative medical treatment reported to have variable success, often not resulting in complete resolution. Surgical management may include the prolonged use of drains and partial or complete surgical removal according to the severity of the lesion with complications including dehiscence as a result of trauma and increased tension in the surgical wound (24). In CB facilities housing large breed dogs, prevention of hygromas should be a priority. In situations where it is safe and feasible to do so while maintaining hygienic conditions, these dogs might benefit from being provided with soft bedding, which will help to slow the progression of the condition by reducing pressure on the olecranon region.

A third abnormality identified was interdigital furuncles. Several dog breeds, including the Chinese Shar Pei, Labrador Retriever, and Bulldog, have been reported to be predisposed to bacterial interdigital furunculosis (22). Bully breeds may be more predisposed to interdigital furuncles, possibly because of the conformation of the paw or the heaviness of the breeds (25, 26). In the current study, 9 of the 10 dogs observed with an interdigital furuncle were bully breeds. Prominent interdigital webbing often forms a callus-like surface known as a false paw pad that can lead to altered distribution of weight-bearing to the interdigital skin (25). The short, bristly interdigital hairs are pushed into the hair follicles, becoming traumatically embedded in the skin. The foreign body fragments of hair shaft rich in keratin trigger an inflammatory response leading to pyogranulomatous inflammation and furuncle formation. Furthermore, these lesions may be further compromised by secondary bacterial infections. Supportive care includes analgesia, weight loss, keeping the interdigital skin clean, and the use of lubricating creams. Secondary bacterial and yeast (*Malassezia* spp.) infections typically require topical and/or systemic antimicrobials (23).

A limitation of the study is that longitudinal data was not collected to better understand the effect the length of time housed on a substrate may have on the development of foot or leg problems. Further humidity and other environmental factors were not considered in this study. These areas should be investigated in future studies.

## 5 Conclusion

Overall, the health of the dogs assessed was maintained on all flooring substrates studied, with most dogs examined having ideal body condition, cleanliness, and low prevalence of abnormal paw or leg conditions. A few conditions were identified, including pododermatitis, hygromas, and interdigital furuncles, where management alterations might result in improved outcomes and welfare states for dogs in CB facilities.

## Data Availability

The raw data supporting the conclusions of this article will be made available by the authors, without undue reservation.
